# Commentary: Containing the Ebola Outbreak - the Potential and Challenge of Mobile Network Data

**DOI:** 10.1371/currents.outbreaks.0177e7fcf52217b8b634376e2f3efc5e

**Published:** 2014-09-29

**Authors:** Amy Wesolowski, Caroline O. Buckee, Linus Bengtsson, Erik Wetter, Xin Lu, Andrew J. Tatem

**Affiliations:** Center for Communicable Disease Dynamics and Department of Epidemiology, Harvard School of Public Health, Boston, Massachussetts, USA; Flowminder Foundation, Stockholm, Sweden; Center for Communicable Disease Dynamics and Department of Epidemiology, Harvard School of Public Health, Boston, Massachussetts, USA; Flowminder Foundation, Stockholm, Sweden; Department of Public Health Sciences, Karolinska Institute, Stockholm, Sweden; Flowminder Foundation, Stockholm, Sweden; Department of Management, Stockholm School of Economics, Stockholm, Sweden; Flowminder Foundation, Stockholm, Sweden; College of Information System and Management, National University of Defense Technology, Changsha, China; Flowminder Foundation, Stockholm, Sweden; Department of Geography and Environment, University of Southampton, Southampton, UK; Fogarty International Center, National Institutes of Health, Besthesda, Maryland, USA

**Keywords:** cellphone, disease model, disease outbreak, ebola, mobility, travel

## Commentary

The ongoing Ebola outbreak is taking place in one of the most highly connected and densely populated regions of Africa (Figure 1A). Accurate information on population movements is valuable for monitoring the progression of the outbreak and predicting its future spread, facilitating the prioritization of interventions and designing surveillance and containment strategies. Vital questions include how the affected regions are connected by population flows, which areas are major mobility hubs, what types of movement typologies exist in the region, and how all of these factors are changing as people react to the outbreak and movement restrictions are put in place. Just a decade ago, obtaining detailed and comprehensive data to answer such questions over this huge region would have been impossible. Today, such valuable data exist and are collected in real-time, but largely remain unused for public health purposes - stored on the servers of mobile phone operators. In this commentary, we outline the utility of CDRs for understanding human mobility in the context of the Ebola, and highlight the need to develop protocols for rapid sharing of operator data in response to public health emergencies.

**Mobility patterns and connectivity in West Africa.**
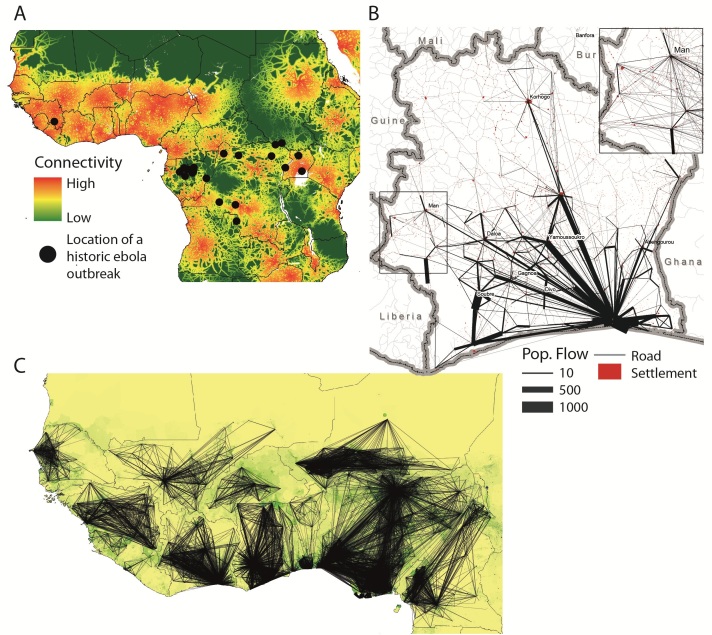
A) Map showing the location of Ebola outbreaks in humans since 1976 (black dots) overlaid on a map of strength of connectivity measured by travel time to the nearest settlement of population 500,000 or more, with dense areas of low travel time indicative of high connectivity. No previously recorded Ebola outbreak has ever occurred in such a densely populated and large area of high connectivity as the ongoing outbreak that began in Guinea; B) Visualization of the flows of 500,000 mobile phone users between the (population-weighted) centres of sous-préfectures in Cote d’Ivoire. The inset highlights the mobility in the western border region (main figure: flows above 20 km with more than 10 average movements per day included, inset figure: flows above 20 km with at least one movement on average per day included); C) Outputs of a within-country mobility model for West Africa built on mobile phone CDRs. The lines show the flows predicted to be greater than 75-95% of the estimated flows per country between settlements for the average number of trips per week and are overlaid on a map of population density (www.worldpop.org.uk).

The rise of mobile phone usage across the past decade, even in the most remote low-income settings, has been astonishing. The global mobile phone penetration rate (i.e. the ratio of active subscriptions to the population) reached 96% in 2014.^1^ In developed countries, the number of subscribers has surpassed the total population, with penetration rates now reaching 121%, while in developing countries it is as high as 90%, and continuing to rise.^1^ Mobile phone networks, also called cellular networks, are composed of cells, i.e. geographic zones around a phone tower. Each communication can be located by identifying the geographic coordinates of its transmitting tower and the associated cell. Mobile call data records (CDRs) detailing the time and associated cell tower of calls and text messages from anonymous users therefore provide a valuable indicator of human presence, and sequences of these data can be used to measure population movements over time, especially when existing mobility data is poor (see Figure S1).^2^^,^ 3

With network operators serving substantial proportions of the population across entire nations, the movements of millions of people at fine spatial and temporal scales can be measured in near real-time and across seasons. Although such data inevitably contain biases due to phone ownership and usage patterns, evidence suggests that these have limited impacts on general estimates of population movement patterns and the relative importance of different travel routes.^4^ The engagement of network operators has resulted in population movement analyses based on CDRs that have been particularly promising for improving responses to disasters 5 ^,^ 6 ^,^ 9 and for planning malaria elimination strategies.^3^^,^ 7 ^,^ 8 ^,^ 19

The benefits of CDRs in the context of the current Ebola outbreak are clear. The rapid spread of the virus within Guinea, Sierra Leone and Liberia, and to Nigeria and Senegal, has been driven by local and regional travel.^18^ Epidemiological models of the spatial spread of Ebola, both retrospectively and for the purposes of prediction, rely on estimates of the volumes and flows of traffic between populations. This allows modelers to assess the likely routes of infected individuals between populations, with imported cases sparking new outbreaks or augmenting local transmission. Since mobility is not only a major driver of the epidemic, but is also likely to shift dramatically in response to the outbreak and be directly targeted by control policies, these estimates are critical. Although the epidemiological data are still highly uncertain and CDRs cannot currently capture cross-border movements, understanding the potential routes of spread of the virus within a country are critical to national containment policies, and will strongly influence more regional spread across borders. Further, the benefits of information on population distributions and mobility for assessment of the implementation of movement restrictions and efficient delivery of interventions, including possible drugs and vaccines, are clear. Detailed aggregated mobility patterns of half a million anonymous phone users on the Orange network 10 in Cote d'Ivoire are shown in Figure 1B, information that would be unobtainable through any other means.

In the absence of operator data from the currently affected countries, we have produced spatial interaction models of national mobility patterns parameterized using CDRs from Cote d’Ivoire, Senegal (made available by Orange in response to the Ebola epidemic^10^), and Kenya (Appendix 1). Figure 1C shows a visualization of the outputs from this initial set of freely available mobility models (for download links and regular updates follow: www.flowminder.org). These models estimate the amount of travel between predefined locations using data on the size of population of the locations and the distances between them (Appendix 1), but do not take into account behavioural changes as the outbreak has progressed and the impact of travel restrictions on population mobility. The models are likely to capture the most important routes of travel and the relative volume of traffic between different populations in the region, but substantial uncertainty remains due to lack of contemporary operator data from different countries in the region and the potential for regional variations in mobile phone usage and ownership patterns.^4^ While the spatial scales of mobility estimates are defined by tower coverage, ranging from hundreds of meters in urban centers to several kilometers in rural areas, understanding travel between population centers is likely to be most critical for planning national and regional containment strategies. Further, the integration of census-derived migration data enables estimation of a wide range of regional cross-border movement patterns, bounded below by international migration data and above assuming borders do not hinder movement (Appendix 1).

Of particular concern, this regional overview of national mobility patterns shows that large areas of West Africa are likely to exhibit much higher population flows than the currently affected areas. Both the mobile operator data from Cote d’Ivoire and the modelled mobility patterns across the region highlight the dominant influence of large population centers, which serve as hubs of national mobility. Several countries in the region are now suspending flights from affected countries, reducing the flow of travel between national hubs. However, rural areas near porous borders remain vulnerable to Ebola importation, and could undermine containment strategies since many of these border areas are likely to be well connected to population centers within their borders. The border between Liberia and Cote d’Ivoire highlights this vulnerability (Fig. 1B).

Despite the value of CDRs in the face of the Ebola emergency, mobile network data is generally very difficult to access due to commercial and privacy concerns. The data contain detailed information on mobile operators’ system designs, their customers, as well as detailed information about individuals’ locations and mobility.^11^ However, for the purposes of responding to epidemics or other public health emergencies, operators may not need to provide access to their complete databases. Since travel between, rather than within, populations is likely to be the most critical information – particularly in the absence of highly spatially resolved epidemiological information – aggregated data on mobility between populations will often be sufficient. Such connectivity matrices are relatively easy for operators to produce themselves, and are much less sensitive than the raw data with regards to regulator requirements for personal privacy as well as commercial competition between operators. Such matrices could potentially be generated routinely for preparedness planning or in near real-time in response to an epidemic.

Careful interpretation of local contexts and data biases are required to generate robust mobility models from mobile phone data, and on-going efforts to validate and improve estimates are crucial.^3^^,^ 12 ^,^ 13 However, the value of these data in the context of a public health emergency like the ongoing Ebola outbreak is undeniable, particularly when integrated with other datasets, as has been done previously for other infectious diseases.^3^^,^ 7 ^,^ 8 ^,^ 14 Mobile operators such as Orange, Safaricom, Digicel, MTC, and Telenor/Grameenphone, who have previously released anonymous network data for public health purposes, deserve credit for actively engaging with researchers to build partnerships to leverage CDRs for public health and development. The continuing spread of Ebola highlights the reality of emerging infections in our increasingly connected world, and we hope that these partnerships can serve as models for operators, researchers, governments, and agencies globally, and in West Africa in particular.

## Competing Interest

The authors have declared that no competing interests exist.

## Appendix 1

**Containing the Ebola outbreak - the potential and challenge of mobile network data: Appendix 1**

 Materials and Methods

 We analyzed a number of existing data sources from national census microdata samples, mobile phone call detail records (CDRs), and spatial population data in order to attempt to better understand intra and international mobility patterns in fifteen West African countries (Benin (BEN), Burkina Faso (BFA), Cote d’Ivoire (CIV), Cameroon (CMR), Ghana (GHA), GIN (Guinea), GMB (Gambia), Guinea-Bissau (GNB), Liberia (LBR), Mali (MLI), Niger (NER), NGA (Nigeria), Senegal (SEN), Sierra Leone (SLE), and Togo (TGO)). Full details are provided below.

 In order to expand the utility of these data sets and predicted mobility patterns in the region, where possible we have made geographic, population, and mobility data freely available at www.flowminder.org and www.worldpop.org.uk.  These estimated and quantified population mobility patterns represent our best estimate of the flows of individuals within West Africa at the time of writing without taking into account travel restrictions or behavioral changes as the Ebola outbreak has progressed.

 Spatial Population Data

* *We obtained population estimates from the WorldPop Project (www.worldpop.org.uk). Settlement locations were obtained from the recently released WorldPop West Africa dataset (www.worldpop.org.uk/ebola). For the administrative unit-based models outlined below, the population totals for each administrative unit were extracted from the WorldPop layers. For the ‘settlement’-based models, firstly Thiessen polygons around each settlement location were constructed and the total population in each from WorldPop layers were extracted. This follows previous analyses for Kenya 4  and was undertaken to ensure that all populations in the region were captured, and that rural populations were not missed.

  Available Mobility Data

** **Overall, movement data in the West Africa region is poor in terms of spatial and temporal resolution, and availability of recent data.  In the majority of countries, the best, freely available source of movement data is census migration data that quantifies movement patterns in the form of change of residence over the course of a year and over large spatial areas.  For two countries in the region (Senegal and Cote d’Ivoire), we analyzed travel patterns from mobile phone call detail records (CDRs). Using these CDRs, we were able to obtain more recent (Figure S1) and finer resolution depictions, both temporally and spatially, of movement within these countries, although in both instances the subset of individuals likely does not form a representative sample of the population.  Mobile phone data: Two mobile phone data sets were provided by Orange Telecom as part of the Data for Development Challenge (D4D).  Call detail records from a random sample of 500,000 anonymous mobile phone subscribers who were active from December 1, 2011 to April 28, 2012 in Cote d’Ivoire.  The user’s location was provided on the subprefecture level (255 total, out of which 237 had at least one mobile phone tower,) of the routing mobile phone tower.  A detailed description of the data can be found in 9 10. Similarly, CDRs from Orange Telecom subscribers in Senegal were provided through an exceptional authorization in support of ebola control efforts.  For one year, January 1 to December 31, 2013, coarse-grained (123 arrondissements) mobility data for 150,000 randomly sampled individuals were available.  For one year, January 1 to December 31, 2013 coarse-grained (123 arrondissements) mobility data for 150,000 randomly sampled individuals were available (for a detailed description of the data, see.^15^ In the Senegal mobile dataset, anonymous phone users were included if they had at least one communication event during more than 75% of the days during 2013. It is possible that users who call more are on average also traveling more as both behaviors are often positively related to socioeconomic status. This could mean that mobility in general is overestimated. Relative connectivity between areas is likely to be less affected.

Additionally an anonymized comprehensive set of CDRs from June 2008 – June 2009 (excluding February 2009) was provided by the leading mobile phone operator in Kenya (92% market share) for individual subscribers (14,816,521) with locations identified at the mobile phone tower level (12,502 in total).^4^ ** This dataset enabled production of finer resolution mobility estimates than from the aggregated datasets outlined above, with parameters showing little differences from the Cote d’Ivoire and Senegal dataset models (see below). These data were aggregated to quantify human travel patterns over the course of the year between 69 Kenyan districts and 692 mapped settlements.

Census microdata samples were obtained from the Integrated Public Use Microdata Series (IPUMS) International online repository (https://international.ipums.org/international/).  Migration data (question phrased as: “*Where did you live last year/5 years ago/10 years ago?”)* were available in a number of the microdata sets for West African countries and were aggregated either to administrative unit level 1 or level 2 (see Table S1 for a summary of the microcensus data used here**.^16^  These are being updated with more recent samples for version 2 data). For a number of countries (BFA, CMR, GIN, MLI, and SLE), international migration data were also available.  Migration data can serve as a proxy for the relative connectivity between admin units and countries**,^17^  however the type of travel (long term migration) is less relevant for the spread of infectious diseases than short-term movements (both temporally and spatially).

**Table S1: Census microdata samples used in constructing the migration-based mobility models outlined here:**

**Country/Year**	**Fraction of census in sample**	**Households**	**Persons** ****	**Census date**	**Smallest geography**
Burkina Faso 2006****	10****	236,206****	1,417,824****	9/23-12-06****	commune****
Cameroon 2005	10	345,363	1,772,359	11/11/2005	arrondissement
Ghana 2000	10	397,097	1,894,133	26/03/2000	district
Guinea 1996	10	108,793	729,071	01/12/1996	prefecture
Mali 2009	10	235,834	1,451,856	14/04/2009	district
Senegal 2002	10	107,999	994,562	N/A	department
Sierra Leone 2004	10	82,518	494,298	04/12/2004	chiefdom

Models of movement

* *Multiple movement models were developed for within country and between country travel patterns using the available data sources described above and pre-existing models.  The gravity model is the simplest spatial interaction model, where the amount of travel (N_ij_) between two locations (i,j) is dependent on their populations (pop_i_, pop_j_) and the physical distance separating them (d(i,j)):

\begin{equation*}N_ij=k ((pop_{i }^{\alpha } + pop_{j}^{\beta } ) / dist(i,j)^{\gamma } \end{equation*}

, where the parameters α, β, κ and γ are fit based on a Poisson distribution**.^17^  Table S2 shows the parameter estimates for the fitted models.

**Table S2: The parameter estimates from fitted gravity models.**

**Locations**	**Intercept (k)**	**Pop From (α** **)**	**Pop To (β** **)**	**Euclidean Distance (γ** **)**	**% Reduction in Deviance**
Cote d'Ivoire (***civ***)	-13.83	0.86	0.78	-1.52	73.28
Senegal (***sen***)	-3.93	0.47	0.46	-1.78	89.73
Kenya – district (***kenya***)	-20.61	1.22	1.22	-2.05	80.06
Kenya - settlement	-6.00	0.66	0.61	-0.67	47.31
Entire IPUMS migration data set (***ipums***)	-23.51	1.13	1.11	-0.95	95.30
IPUMS – BEN (***ipums_country***)	-13.86	0.82	0.79	-0.95	91.29
IPUMS – BFA (***ipums_country***)	-25.32	1.07	1.09	-1.03	60.89
IPUMS – CIV (***ipums_country***)	-15.72	0.90	0.86	-1.18	95.88
IPUMS – CMR (***ipums_country***)	-29.85	1.10	1.51	-0.93	68.33
IPUMS – GHA (***ipums_country***)	-12.68	0.29	1.04	-0.94	66.99
IPUMS – GIN (***ipums_country***)	-29.45	0.99	1.64	-0.69	69.40
IPUMS – GMB (***ipums_country***)	-20.59	1.05	1.01	-1.31	78.00
IPUMS – GNB (***ipums_country***)	-16.98	0.94	0.91	-0.98	91.24
IPUMS – LBR (***ipums_country***)	-16.04	0.90	0.86	-1.04	95.55
IPUMS – MLI (***ipums_country***)	-27.45	1.03	1.25	-0.59	62.05
IPUMS – NER (***ipums_country***)	-6.13	0.56	0.54	-1.09	90.59
IPUMS – NGA (***ipums_country***)	-17.90	0.96	0.92	-0.99	93.57
IPUMS – SEN (***ipums_country***)	-15.20	0.42	1.07	-1.09	68.07
IPUMS – SLE (***ipums_country***)	-54.58	1.67	2.89	-0.51	60.10
IPUMS – TGO (***ipums_country***)	-16.58	0.93	0.89	-1.33	98.61

In prior work 17 a number of gravity models were fit to a more comprehensive mobile phone data set in Kenya.  These existing models were used to also provide estimates on the average number of trips per week between settlements in each country. Gravity models were fit to all the sets of mobile phone CDRs, the entire set of census migration data, and each country’s individual census migration data (Table S2).  For countries missing from each source of data, the estimated parameters from these models were used to estimate amounts of travel.

 Spatial Interaction Models

* *The following sets of models were produced, and Table S2 provides the parameter estimates from the fitted gravity models. Figure S2 shows the predicted ranges of within-country mobility for four of the models.

 ***1.*** ***Ipums***

Gravity model fit to the entire census microdata set

***2.*** ***ipums_country***

Gravity model fit to each country’s census microdata set

***3.*** ***civ***

Gravity model fit to mobility between subprefectures in Cote d’Ivoire from mobile phone CDRs

***4.*** ***kenya***

Gravity model fit to mobility between districts in Kenya from mobile phone CDRs

***5.*** ***sen***

Gravity model fit to mobility between arrondissements in Senegal from mobile phone CDRs

 Figure S2 shows the predicted ranges of within-country mobility for four of the models.

A major component of this work are freely available processed mobility data (when available) and model outputs from gravity models.  Below is a description of the data that are freely available at www.worldpop.org.uk/ebola.

 **1.** **AdminUnits_Within.csv**

****a) All pairs of within country census microdata sublocations

b) Number of trips from the census microdata

c) Population estimates

d) Euclidean distance between sublocation centroids

e) Model predictions from ***ipums***, ***ipums_country***, ***civ***, ***senegal***, and *****Kenya*****

**Table S3: AdminUnits_Within.csv variable descriptions.**

**Variable Name**	**Description**
*from_loc*	Origin location admin unit 1 or 2
*to_loc*	Destination location admin unit 1 or 2
*amt*	Amount of migration reported in the census microdata or modeled amount from [Ref]
*country*	Country ISO code
*from_pop*	Origin population (www.worldpop.org.uk)
*from_x*	Origin centroid, x
*from_y*	Origin centroid, y
*to_pop*	Destination population (www.worldpop.org.uk)
*to_x*	Destination centroid, x
*to_y*	Destination centroid, y
*euc_dist*	Euclidean distance between polygon centroids
*predict_ipums*	Predicted amount of travel from microcensus model (***ipums***)
*predict_ipums_country*	Predicted amount of travel from microcensus model per country (***ipums_country***)
*predict_civ*	Predicted amount of travel from CIV model (***civ***)
*predict_kenya*	Predicted amount of travel from Kenya model (***kenya***)
*predict_sen*	Predicted amount of travel from Senegal model (***sen***)

2.**** **AdmUnits_WBtwn.csv**

a) All pairs of sublocations (including international pairs) from the census microdata

b) Number of trips from the census microdata

c) Population estimates

d) Euclidean distance between sublocation centroids

e) Model predictions from ***ipums***, ***ipums_country***, ***civ***, ***senegal***, and ***kenya***

**Table S4: AdmUnits_WBtwn.csv variable descriptions.**

**Variable Name**	**Description**
*from_loc*	Origin location admin unit 1 or 2
*to_loc*	Destination location admin unit 1 or 2
*from_pop*	Origin population (www.worldpop.org.uk)
*from_x*	Origin centroid, x
*from_y*	Origin centroid, y
*from_loc_adm_id*	Origin location ID (matches labels in AdminUnits_Within.csv)
*from_loc_country*	Origin location country (matches labels in AdminUnits_Within.csv)
*to_pop*	Destination population (www.worldpop.org.uk)
*to_x*	Destination centroid, x
*to_y*	Destination centroid, y
*to_loc_adm_id*	Destination location ID (matches labels in AdminUnits_Within.csv)
*to_loc_country*	Destination location country (matches labels in AdminUnits_Within.csv)
*euc_dist*	Euclidean distance between polygon centroids
*predict_ipums*	Predicted amount of travel from microcensus model (***ipums***)
*predict_civ*	Predicted amount of travel from CIV model (***civ***)
*predict_kenya*	Predicted amount of travel from Kenya model (***kenya***)
*predict_sen*	Predicted amount of travel from Senegal model (***sen***)

3. **MigrationBtwnCountries.csv**

a) Migration from Burkina Faso, Cameroon, Guinea, Mali, and Sierra Leone to other countries from census microdata

**Table S5: MigrationBtwnCountries.csv variable descriptions.**

**Variable Name**	**Description**
from_loc	Origin country
to_loc	Destination country
amt	Amount of migration reported in the census microdata
from_x	Origin centroid, x
from_y	Origin centroid, y
to_x	Destination centroid, x
to_y	Destination centroid, y

4. **CIV_GModel.csv**

a) Predictions from the gravity model (***civ***) of movement between subprefectures based on mobile phone data from Cote d’Ivoire.  The processed amount of travel is also available.

5. **Sen_GModel.csv**

a) Predictions from the gravity model (***sen***) of movement between arrondissements based on mobile phone data from Senegal.  The processed amount of travel is also available. 

**Table S6: CIV_GModel.csv and Sen_GModel.csv variable descriptions.**

**Variable Name**	**Description **
*from_loc*	Origin location admin unit 1 or 2
*to_loc*	Destination location admin unit 1 or 2
*amt*	The estimated mobility from CDR data (provided by Orange Telecom)
*from_pop*	Origin population (www.worldpop.org.uk)
*from_x*	Origin centroid, x
*from_y*	Origin centroid, y
*to_pop*	Destination population (www.worldpop.org.uk)
*to_x*	Destination centroid, x
*to_y*	Destination centroid, y
*euc_dist*	Euclidean distance between polygon centroids
*predict_model*	Predicted amount of travel from country, mobile phone data based gravity model
